# Green approaches for extraction, chemical modification and processing of marine polysaccharides for biomedical applications

**DOI:** 10.3389/fbioe.2022.1041102

**Published:** 2022-12-08

**Authors:** Margarida M. A. Sacramento, João Borges, Fernando J. S. Correia, Ricardo Calado, João M. M. Rodrigues, Sónia G. Patrício, João F. Mano

**Affiliations:** ^1^ CICECO–Aveiro Institute of Materials, Department of Chemistry, University of Aveiro, Aveiro, Portugal; ^2^ Laboratory of Scientific Illustration, Department of Biology, University of Aveiro, Aveiro, Portugal; ^3^ Centre for Environmental and Marine Studies (CESAM), Department of Biology, University of Aveiro, Aveiro, Portugal

**Keywords:** marine-origin polysaccharides, marine biomass, green chemistry, circular economy, sustainability, biomedical applications, blue biotechnology

## Abstract

Over the past few decades, natural-origin polysaccharides have received increasing attention across different fields of application, including biomedicine and biotechnology, because of their specific physicochemical and biological properties that have afforded the fabrication of a plethora of multifunctional devices for healthcare applications. More recently, marine raw materials from fisheries and aquaculture have emerged as a highly sustainable approach to convert marine biomass into added-value polysaccharides for human benefit. Nowadays, significant efforts have been made to combine such circular bio-based approach with cost-effective and environmentally-friendly technologies that enable the isolation of marine-origin polysaccharides up to the final construction of a biomedical device, thus developing an entirely sustainable pipeline. In this regard, the present review intends to provide an up-to-date outlook on the current green extraction methodologies of marine-origin polysaccharides and their molecular engineering toolbox for designing a multitude of biomaterial platforms for healthcare. Furthermore, we discuss how to foster circular bio-based approaches to pursue the further development of added-value biomedical devices, while preserving the marine ecosystem.

## Introduction

The unparalleled biological diversity of the marine realm is an extraordinary source of a plethora of natural macromolecules with higher bioactivity and chemical novelty when compared with those extracted from terrestrial life forms (Zhang et al., 2016), thus making them particularly attractive to be applied in biomedicine and healthcare (Wan et al., 2021; Iliou et al., 2022). Despite the marine environment reconnaissance as an endless diversity of natural compounds, the exploitation of marine biodiversity as a source of high added-value natural compounds is still at its early-stage, thus deserving further comprehensive attention (Vieira et al., 2020). For instance, amongst the world capture of marine species, more than 35% of the total weight has been discarded as by-products of processed marine organisms (Vázquez et al., 2013). Such biomass constitutes a valuable source of a diverse set of novel macromolecules and bioactive products with key properties and functions that extensively surpass those obtained by synthetic means. Furthermore, the inherent renewable character of marine resources will assure new and promising benefits, including the provision of innovative compounds with a lower carbon footprint, as well as a highly valuable alternative to polymers obtained from fossil fuels. However, one should look at a conscious, prudent and sustainable use and management of marine living resources, which implies the reduction of overfishing, pollution and habitat destruction, in order to protect, valorize and secure their survival and maintain the marine ecosystem (Samalens et al., 2022).

**FIGURE 1:**
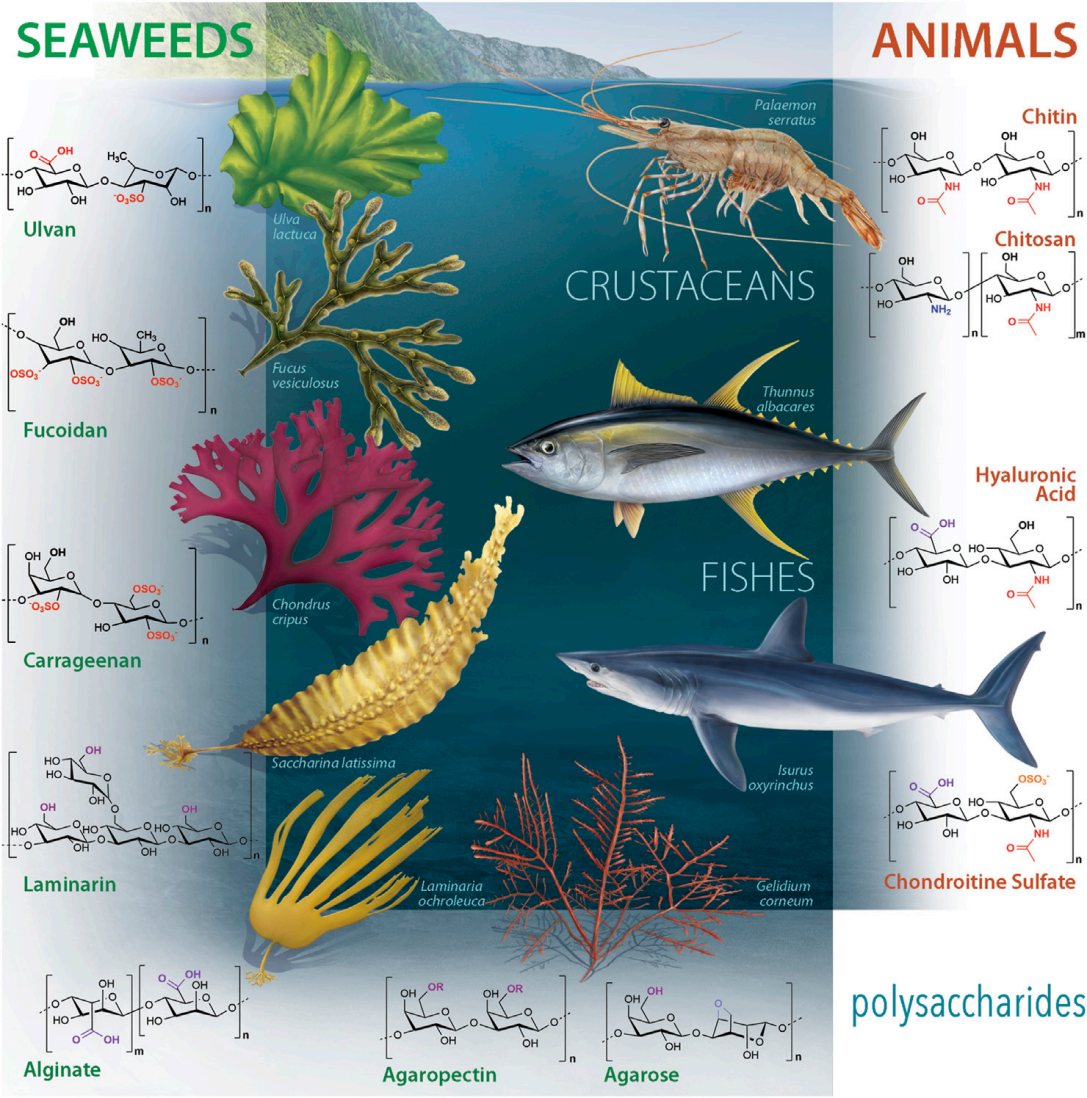
Marine-origin polysaccharide portfolio: chemical structures and representative marine sources.

Among the source of natural compounds found in the oceans, polysaccharides obtained from marine organisms (Figure 1) are currently being investigated for different biomedical applications, namely tissue engineering (TE) (Arokiarajan et al., 2022), drug delivery (Sun et al., 2021) and regenerative medicine (RM) (Mahendiran et al., 2021; Rial-Hermida et al., 2021). The requirements for a material to be suitable for such biomedical applications are biocompatibility, biodegradability and non-immunogenic properties. Besides, marine-origin polysaccharides are readily and widely available, thus enhancing their potential as a low-cost and green strategy for the development of new materials for the biomedical field (Chiellini and Morelli, 2011).

With the aim of maximizing the value of marine by-products towards the development of high value-added biomaterials, this review provides an up-to-date outlook on efficient, cost-effective and environmentally-friendly strategies that have been employed from the isolation of marine-origin polysaccharides up to obtaining the envisage biomedical device. Finally, we highlight some guidelines to facilitate the translation of marine-derived biomaterial platforms to the market and their benefits for society, while fulfilling the United Nations Sustainable Development Goals.

## Green extraction

Most conventional extraction processes (e.g., soxhlet, solid-liquid extraction, or liquid-liquid extraction) are characterized by long extraction times and comprise hazardous acidic and alkali treatments (Ibañez et al., 2012). These conventional extraction methods are also associated with both low selectivity and extraction yields of bioactive compounds. To tackle these issues, the scientific community has been looking for eco-friendly and efficient approaches following the Green Chemistry principles (Ibañez et al., 2012), aimed at supporting scientists and engineers on the design of new materials (Ibañez et al., 2012). Those techniques are mostly based on compressed fluids, including Supercritical Fluid Extraction, Pressurized Hot Water Extraction, and Pressurized Liquid Extraction, also known as Accelerated Solvent Extraction.

Aside from compressed fluids, extraction processes encompassing Subcritical Water Extraction (SWE), Ultrasound-Assisted Extraction (UAE) and Microwave Assisted Extraction (MAE) have also been used to surpass the bottlenecks of the standard extraction procedures, since they could be applied at pilot scale, thus offering the possibility to be integrated into a process chain within a biorefinery. Table 1 summarizes the main features beyond each green extraction technique, as well its advantages and major drawbacks.

**TABLE 1:** Main features of Green Extraction Techniques summarized from (Bruno et al., 2019).

Technique	Extraction principle	Advantages	Disadvantages
Supercritical fluid extraction (SFE)	Solute separation from a solid or liquid matrix using extracting solvents above or near their critical temperature and pressure; carbon dioxide (CO2), alone or combined with another solvent, is the most used supercritical fluid, due to its mild critical conditions (31.1°C and 73.8 MPa). High diffusion coefficient and low viscosity allow the rapid penetration of supercritical CO2 through the matrices pores helping to dissolve the solute like a liquid.	Non-toxic;	SFE efficiency is mostly affected by pressure, extraction temperature and time, density and flow rate of CO2.
		Chemical inertness;	
		Low cost and easy recycling.	
Pressurized liquid extraction (PLE) or accelerated solvent extraction (ASE®)	Enhanced version of liquid extraction;	Reduced extraction time;	Inadequate for thermosensitive compounds;
	Utilization of organic liquid solvents at high temperatures (50–200°C) and pressure (3.5–20 MPa), causing rapid diffusion of solvent, increasing solubility and accelerating the mass transfer of the target compounds.	Less solvent utilization;	Lack of selectivity.
		Increased extraction yield;	
		Suitability of a wider range of solvents.	
Subcritical water extraction (SWE)	Water application under high temperature (100–374°C) and pressure (10–60 bar) in the liquid state, causing a decrease in the viscosity and, an increase in water diffusion and in the extraction of polar compounds.	Reduction of the need of solvent;	N.A.
		Increased extraction rate and yield;	
		Efficient extraction of more polar molecules.	
Ultrasound-assisted extraction (UAE)	Extraction based on the acoustic cavitation phenomenon.	Suitable for thermolabile compounds;	Long sonication times at a higher ultrasound intensity have a negative effect on extraction yield and the crystallinity of some biomaterials.
		Quicker, clean and reproducible.	
Microwave-assisted extraction (MAE)	Extraction based on the temperature increase due to the absorption of electromagnetic radiation (300 MHz to 300 GHz); this increases the pressure inside the cell, due to moisture evaporation, leading to cell wall disruption.	Increase of extraction yield;	Process efficiency affected by microwave power, frequency, sample size, humidity and viscosity, extraction cycles and time, pressure and nature of the solvent.
		Time and energy efficient;	
		Maintenance of the crystalline structure of the extracted compounds.	
Pulse electric field assisted extraction (PEF)	Application of electric pulses to the biomaterial at a pulse amplitude range from 0.1–0.3 kV/cm to 20–80 kV/cm, inducing cell membranes damage, creating pores – electroporation – and facilitating the release of intracellular content.	Non-thermal process, making it suitable for extraction of heat-sensitive compounds;	Extraction yield influenced by electric field intensity, pulse number, material–liquid ratio and NaOH concentration.
		Accelerates the extraction yield and increases the purity of target compounds.	
High hydrostatic pressure extraction (HHP)	Technique based on high pressure application, inducing the charged groups deprotonation and the disruption of weak bonds (e.g. hydrogen, electrostatic, Van der	Proper for temperature sensitive materials;	N.A.
	Waals and hydrophobic bonds) in cellular membranes, increasing cell’s permeability and enhancing the extraction of biomolecules.	Independent of sample size, shape or composition;	
		Better yields and decreased process times due to increase in solvent permeability and enhancement of mass transfer rate.	

This section provides an overview of the extraction methods applied to date to recover the most studied marine polysaccharides, including chitin, chitosan, hyaluronic acid, chondroitin sulfate, ulvan, alginate, fucoidan, laminarin, carrageenan and agarose (Figure 1).

### Chitin and chitosan

Chitin is the second most common polysaccharide in nature, after cellulose, being present in the exoskeleton of animals including crustaceans and shrimps. Chitosan (CHT) is a cationic polymer that is obtained by the deacetylation of chitin (Venugopal, 2011).

Standard processes to extract chitin and CHT usually involve the use of high temperatures combined with strong harsh solvents, which consequently contribute to the generation of hazardous effluents and the quality decrease of the extracted polymers (Nouri et al., 2016). In this regard, some researchers have been focused on finding more environmentally friendly alternatives for their extraction. One of the successfully applied techniques is Subcritical Water Extraction (SWE). To recover chitin from the by-products of shrimp cephalothorax (Espíndola-Cortés et al., 2017), in a 82.2% yield (wt; Table 2), one of the highest chitin content already reported in the form of α-chitin. The optimal conditions for the extraction involved a process time of 30 min at 260°C and a by-product:water ratio of 0.17% (w/w). Moreover, SWE has afforded the complete removal of the protein fraction from the polymeric structure, which has been a bottleneck in the use of chitin due to allergic reactions in sensitive customers (Espíndola-Cortés et al., 2017).

**TABLE 2:** Green extraction techniques applied to recovery polysaccharides from marine raw sources.

Origin	Polysaccharide	Marine Source	Extraction method	Yield(%)±(SD)	REF
ANIMAL	Chitin	Cephalothorax	SWE	82.2	Espíndola-Cortés et al. (2017)
		Litopenaeus vannamei	EE	19.33	Hongkulsup et al. (2016)
	CHT	Metapeneus monodon	MAE	15.25 ± 0.90	Alishahi et al. (2011)
		Penaeus indicus	MAE	19.47	Nouri et al. (2016)
	HA	Aetobatus narinari	EE	0.81 mg/g*	Sadhasivam et al. (2013)
	CS	Raja kenojei	EE	47.44% (w/w)	(Song et al., 2017)
		Tilapia	UAE/MAE	2.513%**	Cheng et al. (2013)
		Fish bones	PEF	6.92 (g/L)	He et al. (2014)
SEAWEED	UL	Ulva meridionalis	MAE	40.4 ± 3.2	Tsubaki et al. (2016)
		Ulva ohnoi	MAE	36.5 ± 3.1	Tsubaki et al. (2016)
	ALG	Saccharina japonica	SWE/DESs	27.21	Saravana et al. (2018)
		Sargassum binderi	UAE	28%	Youssouf et al. (2017)
	FU	Fucus vesiculosus	MAE	18.22	Rodriguez-Jasso et al. (2011)
		Undaria pinnatifida	MAE	N.A.	Quitain et al. (2013)
		Saccharina japonica	PLE	8.23	Saravana et al. (2018)
		Saccharina japonica	SWE/DESs	14.93	Saravana et al. (2018)
	LAM	Laminaria hyperborean	UAE	36.97	Kadam et al. (2015)
		Ascophyllum nodosum	UAE	15.02	Kadam et al. (2015)
	Polysaccharides (FU, LAM, and ALG)	Himanthalia elongata	PLE	54.83%	(Santoyo et al., 2010)
	k-CG	Turbinaria ornata	UAE	50%-55%	Youssouf et al. (2017)
		Kappaphycus alvarezii	SWE/DESs	78.75	Gereniu et al. (2018)
		Kappaphycus alvarezii	DES	60.25 ± 1.10	Das et al. (2016)
	Agar	Gelidium sesquipedale	UAE/EE	10.9–18.2	Li et al. (2021)
	AGR	Gracilaria dura	IL	14 ± 0.5	Sharma et al. (2015)

*- of the dry weight of tissue; **-this value refers to extraction rate.

In another study, chitin was extracted from the shells of the shrimp Litopenaeus vannamei by employing an enzyme from *Streptomyces* griseus. After 3 h treatment, the deproteination percentage was 91.1% (Hongkulsup et al., 2016). Additionally, the chitin recovered by enzymatic extraction had a lower degree of crystallinity than the one retrieved from chemical extraction, thus exhibiting higher solubility properties. The authors concluded that the mild conditions associated to the enzymatic extraction process contributed to the maintenance of the chitin properties, avoiding its degradation.

CHT has been mainly extracted by green techniques like MAE, which holds great promise due to the decrease of the extraction time and increased yield (Table 1). CHT extracted from the fishing processing wastes of shrimps by MAE has showcased a significantly enhanced deacetylation degree yield when compared to autoclave (Alishahi et al., 2011) and alkaline methods (Nouri et al., 2016). Such works have still highlighted the potential of MAE technique to achieve these outcomes in very low reaction times, thus reducing the energy consumption.

### Hyaluronic acid and chondroitin sulfate

HA and CS are part of the glycosaminoglycan (GAG) family and can be extracted from the cartilage, bones and fins of several marine organisms (e.g., whales, sharks, rays, salmon, *etc.*) (Cheng et al., 2013; Sadhasivam et al., 2013; Abdallah et al., 2020). In general, cartilage is the most commonly used source of CS, while the vitreous humor is the main source of HA (Abdallah et al., 2020).

Both HA and CS display interesting physicochemical and biological properties and are widely present in the extracellular matrix (ECM) of connective tissues. Owing to their negative surface charge, they are capable to absorb a large amount of water, thus favouring tissue hydration. In general, HA is known for providing viscoelasticity, lubrification and immune system modulation, while CS displays anti-inflammatory activity, having a central role in the biological processes (Abdallah et al., 2020).

The enzymatic hydrolysis is the most standard applied method for the extraction of GAGs, requiring the use of specific enzymes (e.g., papain, trypsin, pepsin and pronase) to breakdown the protein core and isolate HA and CS macromolecules from other polysaccharide complexes (Sadhasivam et al., 2013; Abdallah et al., 2020). Among the enzymes’ diversity, the most commonly employed to recover CS and HA polysaccharides is papain owing to its highest efficiency. For instance, HA has been extracted from the liver of marine stingray *Aetobatus narinari* (usually discarded in the sea as a waste by food processing companies) through the use of papain (Sadhasivam et al., 2013). The enzymatic degradation can also be coupled with other extraction methods. In this regard, the CS isolation from thornback skate (*Raja clavata*) was carried out by combining the enzymatic degradation approach with chemical hydrolysis through the use of papain in the presence of an alkaline hydroalcoholic solution (Murado et al., 2010).

Although less exploited, other enzymes have also been used for enzymatic digestion. For instance, HA was extracted from the vitreous humor of fish eyes using a protease from *Streptomyces* griseus (Amagai et al., 2009).

Despite being the most standard employed extraction procedure, enzymatic digestion is time-consuming, requiring high energy consumption and several purification steps that lead to low extraction rates. To tackle these issues, the recovery of CS from Tilapia fish species encompassing ultrasonic-microwave synergistic extraction was attempted (Cheng et al., 2013). Several experimental variables were investigated, aiming to establish the optimal extraction conditions. Although being time and energy saving, the extraction yield was low (2.5%). Furthermore, more recently, one attempt to recovery CS from fish bones was carried out by high intensity pulsed electric fields (He et al., 2014). Results have shown that CS content (Table 2) was higher in comparison with the yield values obtained either by enzyme digestion or alkali extraction, holding a great promising strategy to recover bioactive compounds.

### Ulvan

Ulvan (UL) is one of the sulfated polysaccharides extracted from the cell wall of green algae (Chlorophyceae). It can be isolated from seaweed from the genera Ulva and Enteromorpha, that usually have a negative connotation because they are associated with the phenomenon of eutrophication, a detrimental process for the aquatic environment (Chiellini and Morelli, 2011). Therefore, the exploitation of UL can be a remediation strategy towards this problem.

Owing to its variable chemical structure, different molecular weights have been reported for UL (Cunha and Grenha, 2016). Also, the extraction temperature has shown a significant impact in molecular weights variations between ULs extracted from the same species, pointing out that high temperatures are required to extract high molecular weights (Lahaye and Robic, 2007). UL extraction is usually performed using warm water solution (80–90°C) and could be enhanced by the presence of chelating agents (e.g., ammonium oxalate or calcium), acidic or alkaline solutions. Afterwards, UL is generally recovered by precipitation with organic solvents.

UL polysaccharide has also shown to possess different biological properties, such antioxidant (Li et al., 2018), antibacterial (Tran et al., 2018), immunomodulating (Tabarsa et al., 2012), antitumor (Lee et al., 2004), antihyperlipidemic (Pengzhan et al., 2003), antiviral (Aguilar-Briseño et al., 2015) and anticoagulant activities (de Carvalho et al., 2018). The sulphate groups in UL structure are known to be responsible for the aforementioned biological features (Chiellini and Morelli, 2011; Tziveleka et al., 2019).

In the literature, there is not much information regarding the use of green techniques in UL extraction. A recent work has reported the microwave-assisted hydrothermal extraction of UL from two different species, namely *U. meridionalis* and *U. ohnoi* (Tsubaki et al., 2016), attaining maximum extraction yields of 40.4 ± 3.2% and 36.5 ± 3.1%, respectively. Such values were significantly higher than that achieved with conventional hot water extraction (18.7% U. meridionalis) (Tsubaki et al., 2014). Furthermore, microwave-assisted procedure has also allowed to reduce drastically the extraction time (from 3 h to 14 min), without requiring the use of chelating agents and hazardous catalysts. In addition, UL extraction through microwave afforded the control of molecular weights and viscosity by varying the extraction temperature.

### Alginate, fucoidan and laminarin

Alginate (ALG), fucoidan (FU) and laminarin (LAM) are extracted from marine brown algae (Phaeophyceae). Additionally, FU can also be extracted from some marine invertebrates (sea urchins and sea cucumbers) (Lee and Mooney, 2012; Quitain et al., 2013; Cunha and Grenha, 2016). Although LAM is still relatively unexplored, it has recently attracted the attention of the scientific community in the biomedical field due to its low viscosity, cytocompatibility and biocompatibility (Zargarzadeh et al., 2022).

Polysaccharides extracted from brown algae display biological properties, including antioxidant, anticoagulant, antiviral, anti-inflammatory, immunomodulation, and antitumor activities, highlighting their importance in the biomedical field (Saravana et al., 2018). These polysaccharides have been usually extracted from brown algae through several time-consuming extraction approaches, namely hot water, dilute alkali or acid, and various purification and fractionation processes.

Therefore, alternative methods encompassing less extraction times and the use of non-harsh solvents have been investigated, including microwave-assisted extraction (MAE) combined with hydrothermal treatment. One work has investigated a wide range of operational conditions (reaction time, pressure and alga/water ratio), aiming at establishing the best ones that could maximize the FU extraction yield (Rodriguez-Jasso et al., 2011; Quitain et al., 2013). The optimal extraction conditions (pressure 120 psi, 1 min extraction and 1/25 (g ml^−1^) algae/water ratio) have afforded a FU extraction yield of 18.2%. In another work, the microwave–hydrothermal extraction was combined with supercritical carbon dioxide to remove the lipid fraction from the seaweed, prior to FU recovery (Quitain et al., 2013). The hydrothermal process with microwave irradiation has enhanced the degradation of fucoidan into low molecular-weight fractions when compared to conventional heating. Lastly, attempts have been performed to extract fucoidan from Saccharina japonica through PLE, which doesn’t require the use of organic solvent. Although the crude FU yield was the best one achieved (8.2%) comparing to conventional methods (1.8%), microwave-assisted extraction seems to be faster and more efficient than PLE.

Apart from microwave, ultrasound-assisted extraction (UAE) has also been applied as an eco-friendly and time saving technique for the recovery of polysaccharides from brown seaweeds, namely laminarin and alginate (Kadam et al., 2015; Youssouf et al., 2017). In both studies, ultrasound has afforded the reduction of extraction time as well as enhanced the extraction yield over conventional methods.

More recently, one work has reported the synergistic effect of coupling deep eutectic solvent (DES) with subcritical water extraction (SWE) of brown seaweed polysaccharides (FU and ALG) from Saccharina japonica (Saravana et al., 2018). In this regard, the extraction solvent has involved the mixture of different types of DES with water at various concentrations. Alginate and fucoidan yields were different across DES solvents owing to the different electrostatic interaction strength established between these compounds and polysaccharides. Therefore, only one DES solvent (choline chloride:glycerol) has showcased higher extraction efficiency for both polysaccharides. At the optimal experimental conditions, the polysaccharides’ yield obtained in this DES catalyst solvent was at least twice (Table 2) comparing to dilute acid treatment.

### Carrageenan, agar and agarose

Carrageenan (k-CG) and agar (or agar-agar) are marine polysaccharides derived from red seaweed (Rhodophyta) (Cunha and Grenha, 2016).

Owing to their benign nature, ionic liquids (ILs) and deep eutectic solvents (DES) have aroused particular interest as green catalyst in the extraction procedure of carrageenan (k-CG). They can be used either alone or combined with another eco-friendly extraction technique to enhance the polysaccharide yield. For instance, extraction of k-CG from Kappaphycus alvarezii was carried out by using either deep eutectic solvents prepared by the complexation of choline chloride with urea, ethylene glycol and glycerol or by water as standalone extraction media (Das et al., 2016). Interestingly, for the best DES formulation, k-CG yield has achieved the highest value when combined with water (60.3%) due to the higher affinity of DES charges towards k-CG, which are absent in pure water, thus showing lower extraction efficiency (46.9%). In another work, k-CG was extracted from the red marine macroalgae Kappaphycus alvarezii *via* subcritical water extraction (SWE) using ILs as catalyst solvent (Gereniu et al., 2018). Using aqueous SWE alone, the k-CG yield was slightly lower (71.0%) in comparison to SWE combined with ILs (78.8%) (Table 2). Nevertheless, the extraction efficiency of SWE technique was higher either in aqueous or ILs media when compared to conventional alkaline treatments (55.3%).

Concerning agar polysaccharide, one should be mentioned that its structure comprises agaropectin and agarose (AGR); the latter is a neutral and linear polysaccharide that results from enzymatic depolymerization and desulfation of agar (Tiwari and Troy, 2015; Cardoso et al., 2016). Therefore, agarose is usually retrieved by the purification of agar through chromatographic fractionations (Sharma et al., 2015).

Agar is only soluble in hot water (>85°C) and its cooling leads to the formation of thermoreversible gels (Laurienzo, 2010). Therefore, hot-water extraction with alkali pretreatment has been traditionally employed in agar extraction processes. Non-etheless, agar could be leached in hot alkali solution, negatively affecting the extraction yield. To tackle such concerns, an eco-friendly extraction approach of agar was recently explored by combining ultrasonication with two different strategies: i) a controlled alkali pretreatment followed by enzyme-assisted extraction (EAE) and ii) EAE standalone (Li et al., 2021), aiming at generating a synergistic effect by coupling more than one extraction procedure. In fact, EAE has shown to be relevant in both strategies since it has allowed the increase of agar extraction yield by 2 to 6-fold compared to the yield of a non-enzyme method for a total extraction time of 2–3 h. The ultrasonication technique has enabled the reduction of time extraction in 1h, which could be even shorter, at least 30 min, with an alkali pretreatment.

## Chemical and structural modification of marine polysaccharides

Novel materials based on polysaccharides are being intensively pursued through bulk and surface modifications. The chemical and structural modification of marine polysaccharides can improve their solubility, chemical and mechanical stability, processing routes, and the properties of the final polysaccharide-based materials (Siddhanta et al., 2015). Their unique functionalities can be adjusted throughout the coupling of specific functional groups, opening new perspectives for the design of advanced biomaterials. Among the functional groups present in polysaccharides, the hydroxyl groups (–OH) are the most widely explored and chemically modified, although other functional groups have also been used for chemical reactions, including amino (–NH_2_), carboxylic acid groups (–COOH) and aldehydes (–CHO) (BeMiller, 2004).

**FIGURE 2:**
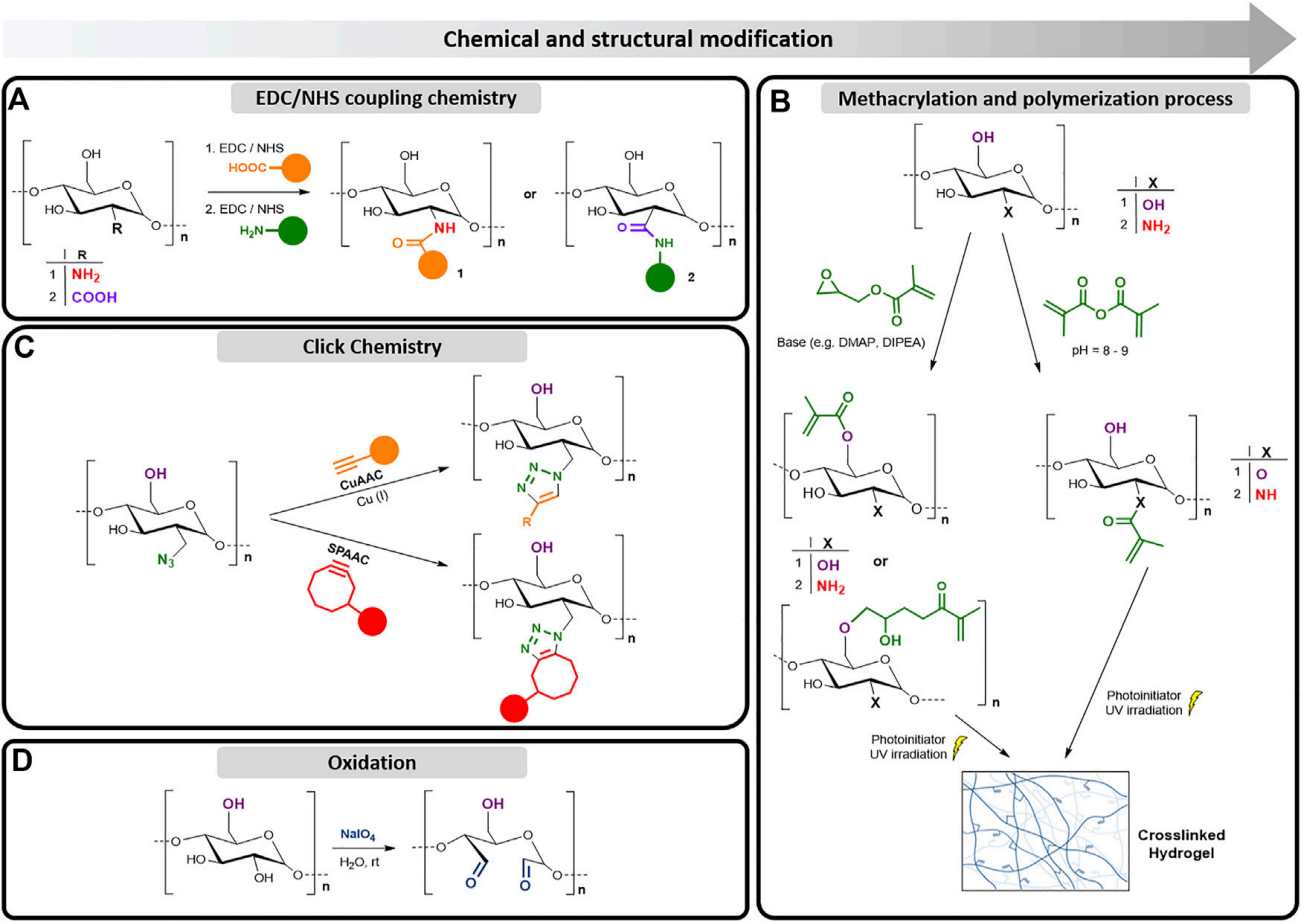
Schematic illustration of major modification strategies applied in polysaccharides based on their functional groups: **(A)** Conjugation to the polysaccharides structure with EDC/NHS coupling chemistry; **(B)** Insertion of methacrylated moieties into the polysaccharide backbone and photo-crosslinking reaction to produce hydrogels; **(C)** Click-chemistry and **(D)** Oxidative cleavage.

The modification of the polysaccharides’ structure through the introduction of acidic, basic, hydrophilic, hydrophobic, or other molecules with specific properties will not change fundamentally the polysaccharide backbone. However, it will unlock advanced modifications required for specific applications, and will change the final properties of the developed biomaterials. The development of novel chemical methodologies is an ongoing research area that is expected to become more important over the years, as the importance on using renewable starting materials and sustainable chemical processes are expanding. The methods described in this section (Figure 2) include nucleophilic reactions of the amines or hydroxyl groups, oxidation of polysaccharides, introduction of nucleophiles into their chains, and activation of functional groups, always taking in account green chemistry approaches or mild conditions commonly employed to modify the marine polysaccharides’ backbone.

### EDC/NHS coupling chemistry

Covalent chemical crosslinking is a widely used technique to enhance physical and chemical stability of the polysaccharide derivatives (Parhi, 2017). In the past decade, 1-ethyl-3-(3-dimethyl-aminopropyl-1-carbodiimide) (EDC) was the most common used chemical agent in carbodiimide crosslinking chemistry and emerged as a popular coupling agent in reactions involving carboxylic acid and amino functional groups, owing to its mild reaction condition, water solubility and self-degradability (Azevedo et al., 2017; Silva et al., 2017; Sousa and Mano, 2017). This graft approach allows to coupling specific molecules with distinct properties, such as methacrylates (Diolosà et al., 2014), catechols (Azevedo et al., 2017; Sousa and Mano, 2017; Costa et al., 2020), among others, into the polysaccharide backbone through a one-step amidation reaction between the amino and carboxylic acid groups. Such coupling reaction is conventionally employed to endow the developing material with application-specific properties (Ye et al., 2017; An et al., 2018; Wang Z. et al., 2018; Sánchez-Téllez et al., 2020). The EDC coupling agent can be used alone or with an additive, usually N-hydroxysuccinimide (NHS), which catalyzes the formation of an amide bond between the carboxyl and the amino groups (Sideris et al., 2016), thus improving the reaction efficiency. Toxicity is absent in the carbodiimide reaction, since EDC is transformed into a non-toxic urea derivative (Park et al., 2002). Other carbodiimide agents like dicyclohexylcarbodiimide (DCC) and N,N′-diisopropylcarbodiimide (DIC) are water-insoluble requiring organic solvents wherein most polysaccharides are non-soluble. Under extreme acid or basic conditions, the EDC/NHS coupling reaction gives rise to the formation of secondary products, thus decreasing the yield efficiency. After the EDC activation of the carboxylic groups, which is more efficient at a pH ∼ 4.5, NHS can be added to the reaction mixture, thus converting the extremely unstable O-acylisourea intermediate into a stable NHS ester. The optimal pH range for the reaction of NHS ester with amine group occurs between 7.2 and 9.0. According to these straightforward reaction conditions, the use of (4-(4,6-dimethoxy-1,3,5-triazin-2-yl)-4-methyl-morpholinium chloride) (DMTMM) showcases major advantages over the conventional EDC/NHS chemistry, since the coupling reaction doesn’t require an accurate pH control for the formation of the amide bonds (D’Este et al., 2014; Borke et al., 2015; Fan et al., 2019) as a result of the coupling reaction between the amino group and the activated ester, produced from the addition of DMTMM to a carboxylate anion (D’Este et al., 2014).

### Methacrylation and polymerization process

Light-initiated polymerization is an important process for the development of novel biomaterials. The insertion of methacrylate groups (MA) into the polymer backbone to be further photo-crosslinked is a widely applied chemical route in natural polysaccharides and used in the fabrication of several biomedical devices, such as 3D hydrogel structures (Bjørge et al., 2018; Martins et al., 2018), cell encapsulation systems (Khademhosseini et al., 2006), and in drug delivery platforms (Jeon et al., 2009). The introduction of photo-crosslinkable moieties into polysaccharide backbones can occur across two distinct functional groups: i) primary hydroxyl groups (Custódio et al., 2016; Martins et al., 2018; Zargarzadeh et al., 2022) and ii) amine groups (Costa and Mano, 2015; Bjørge et al., 2018). The methacrylation reaction *via* hydroxyl and amine groups is carried out through substitution reaction, achieving a higher substitution degree for the latter one due to its nucleophilic strength (higher for amines than hydroxyl groups) (Wang H. et al., 2018; Kolawole et al., 2018). Concerning polysaccharides containing carboxylic acid groups, the metacrylation step is carried out through a coupling reaction with amino groups (Jeon et al., 2009). In such case, the methacrylation degree is commonly lower than the substitution reaction owing to the non-controllable side reactions (Costa and Mano, 2015).

### Click chemistry

Click chemistry (CC) has emerged as a powerful tool for design and synthesis novel polysaccharide derivatives in a more controlled and modular manner (Meng and Edgar, 2016), overcoming major drawbacks from conventional synthetic pathways such as low selectivity, side reactions, low substitution efficiency and low yields. Therefore, click chemistry (CC) is a sophisticated and selective chemical route to covalently link two molecular components *via* simple and high-yielding chemistry. This reaction still fulfills other requirements accounted in the broader Sharpless’ click definition (Kolb et al., 2001), namely both high chemoselectivity and efficiency, wide in scope, simple reaction condition and product isolation as well as fast reaction rate generating inoffensive and easily removable by-products.

The term “click-chemistry” is especially well-known when related to the copper catalyzed azide-alkyne cycloaddition (AAC). The AAC is still the leading technology among CC reactions due to the easy modification and incorporation of azide and alkyne groups into the polysaccharide structure. The application of AAC chemistry in polysaccharides has contributed to generate complex glycoconjugates that allowed the development of new biomaterials exhibiting improved properties and functions (Elchinger et al., 2011), assuring high yields and highly selective products by carbon-hetero bond formation reactions, which results in the formation of a triazole ring unit (Tan et al., 2018). On the other hand, it is also possible to use the biorthogonal click reaction through the strain-promoted azide-alkyne cycloaddition (SPAAC), thus avoiding the use of the cytotoxic copper catalyst (copper-free chemistry). In this scope, we highlight the recent Nobel Prize in Chemistry in which Carolyn Bertozzi has applied such elegant and efficient chemical reaction inside living organisms, without disrupting the normal cell chemistry. Such development has leveraged click chemistry to a new dimension, affording to map cells and track biological processes as well as to improve the targeting of cancer pharmaceuticals (Cioce et al., 2022; Stanczak et al., 2022).

Several reviews have discussed the multiple applications of the (bio)conjugates arise from click-chemistry (Meng and Edgar, 2016; Fantoni et al., 2021; Solberg et al., 2022). Here, we will not summarize the works-to-date on the application of CC to natural polymers but rather focus more precisely on the description of cycloaddition reactions employed in marine polysaccharides, highlighting this powerful chemical binding strategy. Thus, incorporating “click” moieties (e.g., azide or alkyne) into the polysaccharides structure affords either the creation of new materials by “click” crosslinking (Han et al., 2017; Castanheira et al., 2020; Heida et al., 2020; Moody et al., 2020) or the introduction of several functional active molecules for different purposes (Lee et al., 2014; Breger et al., 2015; Duan et al., 2018; Hui et al., 2021).

### Oxidation

Oxidation in polysaccharides can occur at primary and/or secondary hydroxyl groups according to the chemical nature of the mild oxidant agent, thus producing different type of structures (e.g., aldehyde, ketone or carboxylic acid). Typically, the oxidation of primary hydroxyl groups in polysaccharides gives rise to an aldehyde or further to a carboxylic acid group with structural similarity to alginate, while oxidizing secondary alcohols leads to a ring-opening oxidative C–C bond cleavage giving dicarbonyl compounds (Cumpstey, 2013).

Selective oxidation has been attempted using nitrogen dioxide, nitroxyl radical 2,2,6,6-tetramethylpiperidine-1-oxyl (TEMPO) or periodate salts as mild oxidants. Using nitrogen dioxide, primary hydroxyl groups at the C6 position can be oxidized into carboxyl groups. However, oxidative cleavage of polysaccharides may concurrently occur as side reaction, modifying the polysaccharide composition, which is a major drawback (Bragd et al., 2004). In contrast, TEMPO-mediated oxidation features comprise moderate molecular degradation, high selectivity and fast reaction rate (Matsumura and Rajan, 2021). This oxidant agent is suitable for selective oxidation of primary alcohol groups into aldehydes and/or carboxylic acid groups. The selective oxidation of polysaccharide primary alcohols either to the aldehyde or the carboxylic acid levels will rely from the proper selection of oxidizing conditions.

Concerning secondary hydroxyl groups (C-2 and C-3), their oxidation is usually performed by using periodate that causes the oxidative scission of vicinal diols and, ultimately, the formation of dialdehydes or diketones. These groups exhibit high reactivity *via* Schiff base chemistry with compounds bearing amine moieties, thereby widely used for the fabrication of functional biomaterials as hydrogel crosslinking strategy and drug conjugation (Lavrador et al., 2020; Noser et al., 2021).

## Biomaterials processing routes and their biomedical applications

### Fibers, particles and capsules

The development of polysaccharide-based fibers and particles has gathered considerable attention in the biomedical field due to their high surface-to-volume ratio, enhancing loading of cargo as well as efficient nutrient and metabolite transfer and cell-matrix interactions (Kee Jo et al., 2020; Iliou et al., 2022). Advances in precision processing technologies have triggered the design of engineered polysaccharide-based biomaterials with well-defined shapes and sizes, especially aimed at tuning their physicochemical properties for the targeted biomedical application. One of those highly precise and versatile technologies is electrospinning that allows the fabrication of a plethora of polymeric fibrous mesh architectures with controllable fiber diameters, morphologies and arrangements by tailoring electrospinning parameters. These electrospun fiber mats have been widely used as scaffolds in tissue engineering sector (Table 3) due to their inherent fiber morphology that resemble the morphological structure found in native ECM (Silver et al., 2003; Järvinen et al., 2004; Bürck et al., 2013; Wade et al., 2015).

**TABLE 3:** Marine-derived polysaccharides and their processing as fiber/particulate systems for biomedical applications.

Polysaccharide	Chemical modification/Blending	Biomaterial	Application	REF
CHT	CHT-MA	Spheroid-like microgels	Drug Delivery	Bjørge et al. (2018)
		Microcapsules	Cell encapsulation	Costa and Mano, (2017)
	Blended with PEO and crosslink with genipin	Nanofibrous mats	Scaffold for TE	Li et al. (2015)
	Blended with PEO	Nanofibrous mats	Drug delivery	Amiri et al. (2020)
			Antibacterial wound dressing	
HA	Blended with PEO	Nanofibrous mats	Drug delivery	Ahire et al. (2017)
			Coating prosthetic implants	
	HA-AdNor crosslinked under UV light	Injectable microgels	Myocardial tissue regeneration	Mealy et al. (2018)
	HA-MA	3D microgels	Prostate-to-bone tumor model	(Antunes et al., 2019)
CS	Blended with PVA; crosslink with glutaraldehyde	Nanofibrous mats	Drug delivery	Guo et al. (2016)
			Regenerative medicine	
	Blended with gelatin and PCL	Nanofibrous mats	Blood vessel repair	Kong et al. (2021)
ALG	Blended with PEO and Triton X-100 or Pluronic F-127; Physical crosslink (CaCl2)	Nanofibrous mats	Drug delivery	Kyzioł et al. (2017)
			Regenerative medicine	
			Wound healing	
	Multilayer assembly with CHT	Microcapsules	Bone TE	Nadine et al. (2022)
				Bjørge et al. (2022)
				Nadine et al. (2019)
	Physical crosslink (CaCl2)	Microgels	Modular units for TE	Neto et al. (2016)
FU	Blended with CHT and PVA; crosslink under glutaraldehyde vapor phase	Nanofibrous mats	Vascular TE	Zhang et al. (2017)
LAM	Modified with either alkyne or hydrazide groups for triazole crosslinking	Microparticles	Drug Delivery	Castanheira et al. (2020)
	LAM-MA; particle surface functionalization with antibodies	Microparticles	Cell sorting platforms	Martins et al. (2018)
			Injectable scaffolds	
			Modular units for large scaffolding constructs	
k-CG	Blended with CHT and PCL	Nanofibrous mats	Bone TE	Vargas-Osorio et al. (2022)
			Wound healing	
	Carboxyl k-CG blended with PVA and thermal crosslinking	Nanofibrous mats	TE	(C Madruga et al., 2021)

Despite the attractiveness of marine polysaccharides for producing biocompatible electrospun scaffolds, electrospinning of polysaccharide pristine solutions has proven to be challenging since they have poor solubility in common volatile organic solvents as well as low molecular weight, thus hampering stable fiber formation through conventional electrospinning methods. Likewise, marine polysaccharides are often combined with synthetic water-soluble biopolymers, including polyvinyl alcohol (PVA), polyvinyl pyrrolidone (PVP) and polyethylene oxide (PEO), thus providing the desired electrospinnability for continuous fiber formation (Toskas et al., 2011; Sousa et al., 2015b; Guo et al., 2016; Ahire et al., 2017). In particular, the use of electrospinning aqueous medium instead of the harsh organic standard solvents has rendered this technique more attractive not only to produce fully biocompatible tissue-engineered scaffolds, but also to load hydrophilic bioactive compounds that could be intolerant to organic solvents. Antibiotics and antibacterial agents are the most representative hydrophilic biocides that have been successfully blended with water-soluble marine polysaccharides to produce drug-loaded fibers with controlled release profile, thus enhancing wound healing performance (Guo et al., 2016; Ahire et al., 2017; Kyzioł et al., 2017; Amiri et al., 2020). However, in the blending electrospinning method, the drug is mostly localized on the electrospun fiber surface, giving rise to an obvious initial burst release. Rather than blending electrospinning, another spinning method that enables the development of drug loaded polysaccharide fibers is coaxial electrospinning wherein core-shell fibers are formed. Drugs encapsulated in the core layer have exhibited a gradual drug release, while being protected from direct exposure to the biological environment by the polymer shell (Hu et al., 2014). Drugs can also be loaded within both core and shell layers, endowing the electrospun fibers with dual drug release profile. Electrospun core-shell fibers with Nylon-6 as the inner core and CHT/polyethylene oxide as the outer shell were loaded with different antibacterial drugs, yielding a binary antimicrobial system with spatial control release (Keirouz et al., 2020).

Apart from water-based solvents, other green spinning solvents (Avossa et al., 2022), namely formic acid, acetic acid, dimethyl sulfoxide, or a combination thereof, have also been employed to produce electrospun fibers based on CHT (Li et al., 2015; Amiri et al., 2020; Keirouz et al., 2020; Vargas-Osorio et al., 2022), which is soluble in concentrated organic acids. Despite the acid character of those solvents, resulting fiber mats have showcased no cytotoxic effects on cellular responses, thus being suitable to be applied as tissue engineered scaffolds (Li et al., 2015; Kong et al., 2021; Vargas-Osorio et al., 2022). Non-etheless, marine polysaccharide fiber mats generated in aqueous conditions have limited mechanical stability in water-based media, losing their fibrous morphology over time. Therefore, polysaccharide fiber mats have been crosslinked during (*in-situ*) or after (post) electrospinning process, thus enabling the formation of stable hydrogel-based nanofibers. Moreover, crosslinking treatments also contribute to a sustained and prolonged drug release, thus preventing an initial burst release.

**FIGURE 3:**
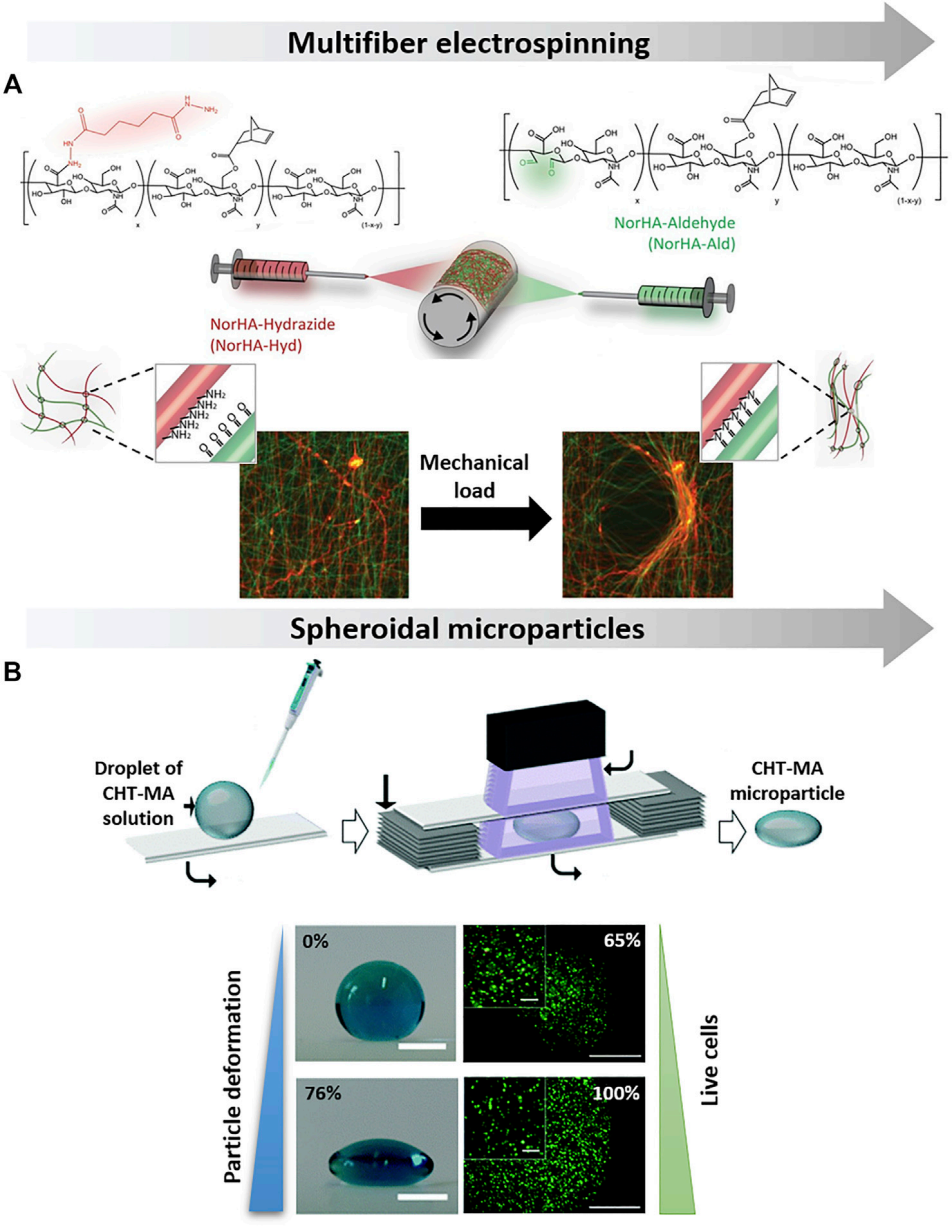
**(A)** Multifiber electrospinning to fabricate fibrous hydrogel networks with complementary chemical moieties (aldehyde and hydrazide groups) that form covalent bonds under mechanical. Adapted from ref (Davidson et al., 2020). with permission from John Wiley & Sons, Copyright 2019 WILEY-VCH Verlag GmbH & Co. KGaA, Weinheim; **(B)** Production of spheroid-like microgels by using superhydrophobic surfaces. Adapted from (Bjørge et al., 2018) with permission from American Chemical Society, Copyright 2018.

Although glutaraldehyde has been reported as the most commonly used crosslinker agent (Guo et al., 2016; Zhang et al., 2017), its hazardous nature could impair scaffold transplantation in tissue engineering scenarios. In this regard, research efforts have been focused on the use of less cytotoxic and more environmental friendly crosslinking strategies, encompassing benign crosslinkers (e.g., genipin, enzymes, EDC alone or with NHS) (Gruppuso et al., 2022), polysaccharide complexation (Chen et al., 2021), thermal treatment (C Madruga et al., 2021) or UV-light exposure (Kianfar et al., 2019). While these green methods require the use of catalysts or external energy inputs for post-crosslinking electrospun fibers, a fast and one-step fiber drawing and crosslinking approach was recently proposed in the form of multiple fibrous hydrogel populations (Figure 3A). These fibers comprised hyaluronic acid-based polysaccharide functionalized with complementary chemical moieties (e.g., aldehyde and hydrazide groups) that were able to form covalently hydrazone bonds within minutes, when brought into contact under mechanical load (Davidson et al., 2020). The combination of multifiber electrospinning with complementary dynamic chemistry has enable the development of fibrous hydrogel networks with mechano-sensitive properties, emulating both structural and mechanochemical features of the native ECM.

Polysaccharide-based particulates, namely microparticles, microgels and microcapsules, have also been broadly applied as individual drug carriers and cell culture platforms (Table 3). Different environmentally friendly strategies have been employed to fabricate spherical polysaccharide-based particulates, spanning from conventional emulsion techniques, droplet microfluidics and electrospraying to superhydrophobic surfaces. The innate presence of functional groups on marine polysaccharides affords specific chemical modification that could be synergistically combined with the aforementioned processing technologies to give rise to advanced particulate systems. Recently, biodegradable LAM-based microparticles were produced *via* triazole crosslinking between alkyne-modified LAM and hydrazine-modified LAM through standard microemulsion (Castanheira et al., 2020). Marine polysaccharide microparticles could also be functionalized with specific antibodies for selective recruitment of different cell types from a mixed cell population (Custódio et al., 2015; Martins et al., 2018). Beyond cell sorting applicability, these platforms could also function as microcarriers for cell expansion to be further applied in TERM either as injectable scaffolds or as modular units for large-scale scaffolding constructs.

Other particulate systems that have also been gained momentum as injectable platforms and building blocks for bottom-up TE approach are microgels, where different populations could be combined into a single granular structure, affording multiple functionalities (Mealy et al., 2018). Micropatterned superhydrophobic surfaces have emerged as one promising eco-friendly and cost-effective approach for high-throughput fabrication of marine polysaccharide-based microgel particles and microcapsules, with multiplex shapes and sizes (Neto et al., 2016; Nadine et al., 2020). Also, standalone superhydrophobic surfaces have afforded the fabrication of core-shell and multicompartmental microgels comprising a photo-crosslinkable CHT outer layer and an ALG core, which could be further liquefied giving rise to a liquid microenvironment that enhanced nutrient up-take and disposal of waste products by encapsulated cells (Costa and Mano, 2017). Non-modified CHT and ALG polysaccharides have also been used as layer-by-layer (LbL) building-blocks in the fabrication of either hollow (Ribeiro et al., 2018) or liquefied multilayered capsules (Nadine et al., 2019, 2022; Bjørge et al., 2022), which could be used for tailoring cellular internalization and bone repair, respectively. Recently, the production of marine polysaccharide-based particulate systems through superhydrophobic surfaces expanded beyond the standard spherical-shaped ones. In this regard, droplets of the precursor microgel were squeezed between two superhydrophobic surfaces separated with spacers with different height (Figure 3B), and then photo-crosslinked to maintain the acquired spheroidal shape after “de-sandwiching” (Bjørge et al., 2018). Those spheroid-like microgels have showcased an improved viability of encapsulated cells due to enhanced nutrient diffusion to the core as well as faster drug release rate from the polymer network.

### Membranes and films

Marine-origin polysaccharides have also been widely used either alone or in combination with other (bio)polymeric materials to prepare membranes, denoting fine-tuned properties and functions at the nanoscale, by resorting to green processing methodologies. Solvent casting/evaporation, LbL assembly technology, and polyelectrolyte complexation have been among the most commonly used methodologies to process biocompatible marine polysaccharide-derived self-standing membranes (Table 4) of the desired size and geometry and without the need for specialized equipment to be used in a wide range of biomedical applications.

**TABLE 4:** Membrane-shaped biomaterials based on marine-derived polysaccharides for biomedical applications.

Polysaccharide	Chemical modification/Assembly	Biomaterial	Application	REF
CHT	Solvent casting and glutaraldehyde crosslink	Membrane	Drug/therapeutics delivery	da Silva et al. (2008)
	Solvent casting blended membrane with soy protein crosslinked with glutaraldehyde	Membrane	TE	Silva et al. (2005)
	Blended membrane with aloe vera crosslinked with genipin	Membrane	Wound dressing	Silva et al. (2013)
	Blended membrane ionically crosslinked with κ-CG and PVA	Hydrogel membrane	Wound dressing	Khaliq et al. (2022)
	Blended membrane ionically crosslinked with CS	Film	Drug/therapeutics delivery	Hashizume et al. (2016)
	Blended membrane ionically crosslinked with CS	Film	TE	Iijima et al. (2017)
	Polyelectrolyte complexes with ALG	Saloplastic membrane	Soft TE; Wound dressing; Drug/therapeutic delivery	Costa et al. (2015)
		Membrane		Wang et al. (2002)
		Membrane		Hardy et al. (2018)
	Polyelectrolyte complexes with CS	Membrane	Cartilage TE	Rodrigues et al. (2016)
HA	Polyelectrolyte complexes with CHT	Free-standing membrane	TE	Larkin et al. (2010)
CS	Genipin covalent crosslink	Nanopatterned membrane	TE	Sousa et al. (2017)
ALG	(Gil et al., 2015) LbL with CHT	Freestanding film	Drug/therapeutics delivery	Caridade et al. (2013)
	LbL with CHT followed by genipin crosslink	Freestanding membrane	TE	Silva et al. (2014)
	LbL followed by EDC/NHS crosslink	Freestanding membranes	Bone TE	Caridade et al. (2015)
	LbL followed by EDC/NHS crosslink	Quasi-3D freestanding membranes	TE	Martins et al. (2017)
	Covalent crosslink with genipin	Shape memory membranes	Implantable devices for biomedical applications	Borges et al. (2015)
	LbL with CHT followed genipin crosslink and physical crosslink (CaCl2)	Shape memory membranes	Biomedical and biotechnological applications	Silva et al. (2016)
	LbL with CHT incorporating MNPs and further genipin crosslink	Freestanding membranes	Biomedical applications	Gil et al. (2015)
	LbL with CHT and HA-dopamine	Freestanding multilayer membranes	Skin wound healing	Sousa et al. (2018)
Agar	Films plasticized with DESs mixture of choline chloride and citric acid (1:1)	Films	Food packaging	Galvis-Sánchez et al. (2016)

Over the last 2 decades, native and crosslinked wholly marine polysaccharide and blended membranes have been produced at a fast pace by solvent casting followed by solvent evaporation and their physicochemical, structural and biological properties studied (Silva et al., 2005; Wu et al., 2010; Yang et al., 2016), revealing that the crosslinking triggers the preparation of stiffer membranes with enhanced cell functions (Silva et al., 2013; Khaliq et al., 2022). Due to their physicochemical, structural and biological properties, these membranes have shown to be promising biomedical devices for the loading and sustained release of drug/therapeutic agents (da Silva et al., 2008; Murata et al., 2010) and as wound dressings (Silva et al., 2013; Khaliq et al., 2022). Moreover, hot press techniques, namely thermo-compression molding has been used to process marine polysaccharide-based composite biofilms/membranes, denoting good mechanical strength and swelling behavior for sustained drug release (Hashizume et al., 2016), controlled molecular permeability (Sagawa et al., 2022), and cell adhesion and proliferation (Iijima et al., 2017). Furthermore, due to their cost-effective and ease of scalability, they can be easily translated into industrial applications. However, the composite films produced with CHT *via* either solvent casting or thermo-compression molding resort to common organic acids for enabling its solubilization. As such, the scientific community has been looking for benign solvents to enable the production of biofilms. In particular, deep eutectic solvents have emerged as alternative green plasticizers for the sustainable production of more homogeneous, compact, and environmentally friendly marine polysaccharide derived composite biofilms/membranes, denoting improved mechanical properties and flexibility (Sousa et al., 2014; 2015a; Galvis-Sánchez et al., 2016, 2018; Zdanowicz et al., 2018). Although such biofilms have been mostly processed for food packaging applications, they hold great potential for being applied in biomedical applications, particularly as wound dressings. Still, the fact that high temperatures are employed in the membranes’ production process poses a major challenge to work with sensitive macromolecules susceptible to thermal degradation and that would benefit from alternative processing strategies under physiological temperature. In this regard, compact biofunctional polyelectrolyte complexes membranes encompassing two oppositely charged marine polysaccharides have been produced at physiological temperature by polyelectrolyte complexation, in the presence of salt, followed by sedimentation and temperature-assisted solvent evaporation for compaction. In particular, microsized soft compact saloplastic membranes encompassing CHT/ALG and CHT/CS have been produced and their physicochemical and biological properties studied aiming for being used as drug/therapeutic carriers and adhesives for soft tissue engineering/regeneration (Wang et al., 2002; Costa et al., 2015; Rodrigues et al., 2016; Zhu et al., 2017; Hardy et al., 2018). It was found that their physicochemical and biological properties could be easily tailored to meet the tissues to be regenerated by playing with the polyelectrolyte types, assembly conditions (e.g., pH, ionic strength, temperature, concentration) and compaction strength.

The production methodology can be easily scaled-up and at a faster pace than the classical solvent casting and LbL assembly methodology due to the high concentration of the polyelectrolyte complexes at the beginning of the drying step. Compact membranes denoting improved adhesive strength to soft tissues at physiological conditions have also been produced by polyelectrolyte complexation and compaction by resorting to hybrid synthetic/natural polyelectrolyte complexes, showing great promise as biomimetic sealants for the regeneration of soft biological tissues (Costa et al., 2019).

**FIGURE 4:**
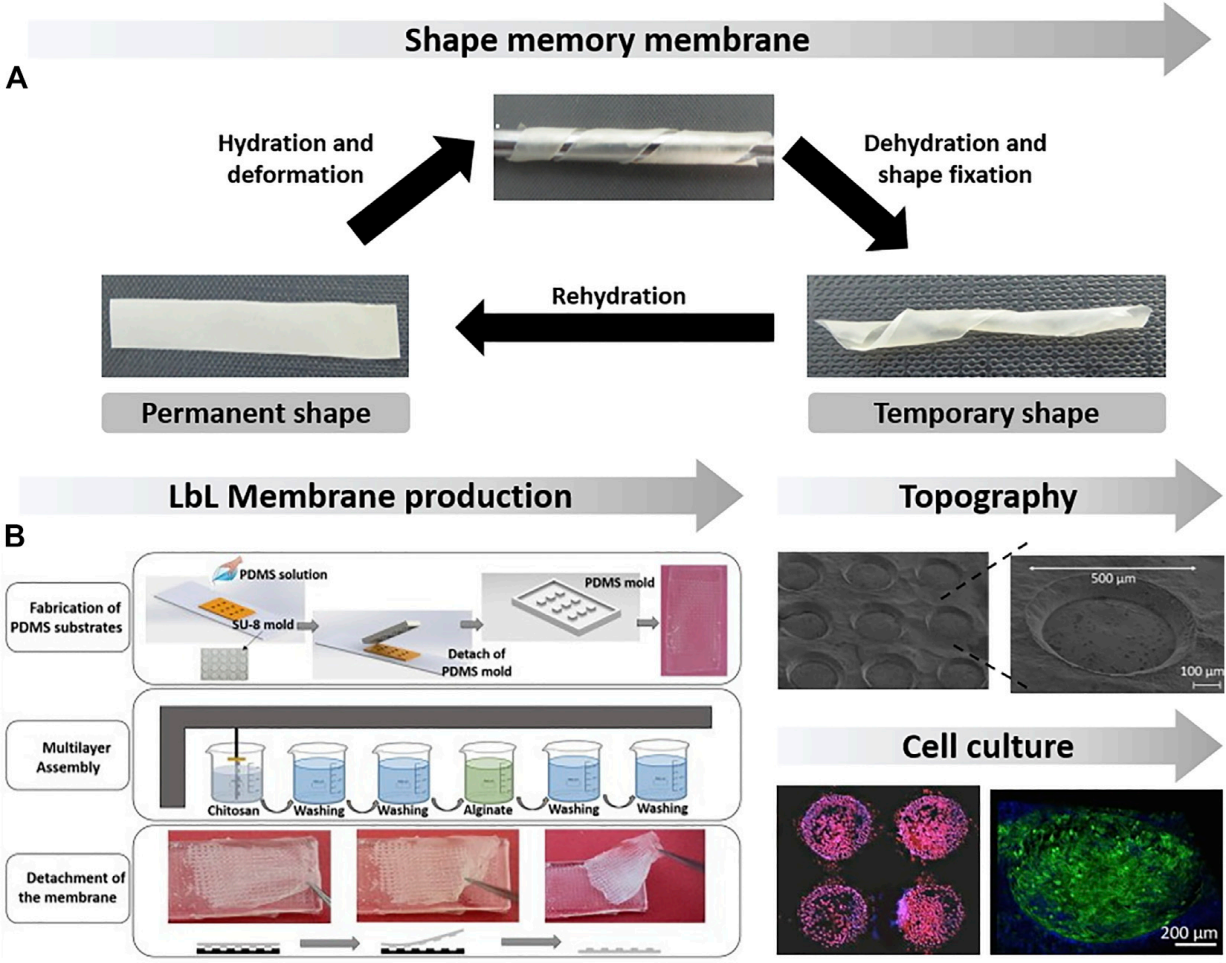
**(A)** Shape memory behavior of (CHT/ALG)100 freestanding multilayered membranes induced by hydration. Reproduced from ref (Borges et al., 2015). with permission from John Wiley & Sons, Copyright 2015 WILEY-VCH Verlag GmbH & Co. KGaA, Weinheim; **(B)** Freestanding multilayered membranes patterned with micro-wells, wherein cells tended to colonize preferentially. Adapted from (Martins et al., 2017) with permission from Elsevier, Copyright 2017.

Over the last 2 decades, we have witnessed to the emergence of the LbL assembly technology as a green bottom-up assembly methodology to process multilayered films/membranes, under mild conditions, in entirely aqueous solutions and on virtually any type of substrate for biomedical applications (Borges and Mano, 2014). The LbL assembly process is a rather time-consuming methodology when compared with the aforementioned methodologies but enables the incorporation of multicomponents and, thus, multifunctionalities in a single membrane and a better control in thickness. Smooth flat hydrophobic and hydrophilic non-patterned or patterned inert templates have been used to produce biocompatible multilayered films, with well-defined structures, compositions, and thicknesses at the nanoscale by repeating their alternate immersion in aqueous solutions of at least two oppositely charged marine polysaccharides *via* dip-assisted LbL assembly methodology (Larkin et al., 2010; Caridade et al., 2013, 2015; Silva et al., 2014; Martins et al., 2017; Sousa et al., 2017). In the end of the assembly process, the multilayered films are easily detached into robust and easily handled free-standing multilayered membranes whose nanotopography recreates the substrate’s features. For instance, CHT/ALG multilayered films were assembled on hydrophobic poly (propylene) substrates and further detached, without any post-processing step, into free-standing membranes by playing with the solution pH, polyelectrolyte concentration and number of adsorbed layers (Caridade et al., 2013). These membranes demonstrated to be stable in physiological media and enabled a faster permeation of model drugs, thus holding great potential for being used as reservoir of therapeutic agents. Furthermore, C2C12 myoblast cells adhered preferentially onto the ALG-ended membranes, thus being potentially used as wound dressings for muscle tissue engineering strategies. This work launched the foundations of several studies encompassing either solely marine polysaccharides or hybrid multilayered systems and further covalently crosslinked aiming to develop free-standing membranes with increased stiffness and better cell response (Silva et al., 2014; Caridade et al., 2015; Gil et al., 2015). In this regard, CHT/ALG freestanding membranes chemically crosslinked with EDC enabled the proliferation and differentiation of C2C12 myoblasts into myotubes, a process whose extent can be easily modulated by playing with the concentration of EDC. Furthermore, the membranes with the highest concentration of EDC demonstrated to be osteoinductive *in vivo*, thus holding great promise for bone repair (Caridade et al., 2015). The effects of either hydration or ionic cross-linking have also been demonstrated as contributing to the development of smart self-sustained membranes denoting adhesive properties and shape memory capability (Figure 4A) to be used as implantable biomaterials *via* minimally invasive surgery (Borges et al., 2015; Silva et al., 2016). In addition, bioinspired free-standing membranes functionalized with catechol moieties have shown enhanced mechanical strength, cellular adhesion and proliferation, and adhesive properties for being used as bio-adhesive patches for wound healing and general surgery applications (Scognamiglio et al., 2016; Sousa et al., 2018; Moreira et al., 2020).

Besides, enzymatic-sensitive polysaccharide-based freestanding membranes have been developed as smart carrier vehicles for the controlled release of bioactive agents in tissue engineering and regenerative medicine strategies (Cardoso et al., 2016). Such strategy was also recently applied to produce protein-loaded mucoadhesive buccal patches for sublingual drug delivery (Paris et al., 2021). Those patches consisted on biodegradable freestanding membranes comprising CHT and HA multilayers, in which the CHT outer layers afforded mucoadhesion owing to the electrostatic interaction of CHT positive charges with negative glycoprotein acid residues from the mucosa surface. Through pH changes, protein rich-ovalbumin was efficiently loaded into the freestanding LbL membranes, producing bioactive patches. Upon sublingual administration in a mouse model, the protein delivered by bioactive patches was detected into the sublingual epithelium 10 min after patch administration, while remaining in the mouse mouth for at least 30 min, even after the enzymatic degradation of the membrane. Such delivery system could open a promising alternative to oral administration, affording a sustained drug release profile when compared to sublingual liquid administration wherein the liquid drug formulation was quickly washed-out in the saliva and poorly delivered at mucosal sites.

More recently, flat patterned templates have gained momentum due to the need to produce bioinstructive patterned freestanding membranes that could recreate not only the structural but also the topographical features of highly aligned tissues, such as bone or nervous tissue, enabling a better regulation of cell functions. Micro-patterned polydimethylsiloxane substrates have been used to produce (CHT/ALG)100 free-standing membranes denoting a well-defined array of geometrical features at the microscale in which osteoblast-like cells tended to colonize preferentially (Figure 4B), holding great promise as micro-reservoirs of therapeutic agents or as cell carriers in bone tissue regeneration (Martins et al., 2017). More recently, nanogrooved CHT/CS freestanding membranes assembled onto nanopatterned polycarbonate substrates guided C2C12 myoblast cells’ alignment along the nanopattern direction and triggered their differentiation into myotubes (Sousa et al., 2017). Such an approach is of high value-added for muscle tissue regeneration but could also be extend for the regeneration of other tissues whose cells respond to topographical cues, such as neuronal cells, thus potentially enabling the regeneration of the nervous system.

### Hydrogels and 3D constructs

Hydrogels have been one of the bioengineering platforms highly explored in the biomedical field to mimic and restore the native tissues’ functionalities. They offer similarity to the ECM, high-water content, mechanical support for cellular activities, and the opportunity to incorporate different molecules (e.g., drugs, cytokines or growth factors). Owing to their structural similarity to ECM compounds, marine polysaccharides have also been extensively employed as precursor building blocks in the hydrogels’ design either in the form of injectable systems or as implantable 3D constructs (Table 5).

**TABLE 5:** Hydrogel-derivatives processed from marine for biomedical applications.

Polysaccharide	Chemical modification/Blending	Biomaterial	Application	REF
Chitin	Epichlorohydrin (ECH) crosslink	Hydrogel	Drug delivery	Shen et al. (2016)
CHT	N.A.	Ionogel [bmim][Ac] (microspheres)	Biomedical applications	Liu et al. (2012)
	N.A.	Ionogel [bmim][Ac]	Skin TE	Silva et al. (2012)
	Carboxymethyl CHT-MA	Ionogel	Spinal cord injury treatment	Wang et al. (2022)
	Supramolecular bonds	Hydrogel (injectable)	Tissue regeneration and cell-based therapies	Oliveira et al. (2018)
	N.A.	Hydrogel	Wound healing (in vivo)	Gan et al. (2018)
	Carboxyethyl CHT	Scaffold	Tissue regeneration	Carlos et al. (2021)
	Genipin crosslink	Sponge	Bone regeneration	Sukul et al. (2021)
	CHT-MA	Hydrogel	TERM	Costa et al. (2022)
	CHT-DOPA and Genipin crosslink	Hydrogel (injectable)	Regenerative medicine and electronic devices	Azevedo et al. (2017)
HA	HA-Ad, HA-CD and HA-AdNor	Hydrogel (injectable)	Biomedical applications where vessel patterning is important	Hoon Song et al. (2018)
	HA-MA	Hydrogel	Cartilage tissue regeneration (in vivo)	Deng et al. (2019)
	Enzymatic crosslinking of HA-g-Dex– tyramine (TA) with HRP using H2O2	Hydrogel (injectable)	Cartilage TE	Jin et al., (2010)
	HA adipic acid dihydrazide (HA-ADH)-MMP	Hydrogel	Diabetic wound healing (in vivo)	Tokatlian et al. (2015)
	HA-MA	Hydrogel (injectable)	Periodontal wound heling	Babo et al. (2017)
	Hydrazide-functionalized HA (ADH-HA) and HA-ALD	Hydrogel (injectable)	Endodontic regeneration	Silva et al. (2018)
	Acrylated HA with RGD (HA-AC-RGD)	Hydrogel	Gene delivery	Villate-Beitia et al. (2018)
	HA modified with zein	Colloidal gels	Biomedical applications	Freitas et al. (2020)
	HA-dopamine	Adhesive hydrogel	Minimally invasive cell therapy	Shin et al. (2015)
	HA-MA and HA-ALD	Adhesive hydrogel	Cartilage regeneration	Chen et al. (2021)
CS	Enzymatically crosslinking of CMP-TA and CS-TA with HRP using H2O2	Hydrogel (injectable)	Cartilage TE (in vivo)	Chen et al. (2016)
	N.A.	Scaffold	Articular cartilage repair	Zhou et al. (2017)
	CS-MA	Hydrogel	Bone tissue regeneration	Kim et al. (2017)
	N.A.	Scaffold	Chronic wound healing	Sharma et al. (2019)
	Freeze-drying of CS solution with multi-walled CNTs (MWCNTs)	Scaffold	Nervous tissue repair	Serrano et al. (2014)
	N.A.	Ionogel [Hmim][HSO4]	Biomedical and Pharmaceutical applications	Nunes et al. (2017)
UL	UL-ALD	Hydrogel	Chronic diabetic wound healing (in vivo)	Ren et al. (2022)
	N.A.	Scaffold	Bone TE	Kikionis et al. (2021)
	UL-Acrylate-Pnipaam	Hydrogel	Biomedical applications	Morelli et al. (2016)
	Crosslinked UL/GEL	Sponge-like scaffold	Bone TE	Tziveleka et al. (2020)
	N.A.	PEC Scaffold	Bone TE	Dash et al. (2018)
ALG	Physical crosslink (CaCl2)	Hydrogel (injectable)	TERM	Sousa et al. (2021)
	N.A. (solvent casting)	Hydrogel	Wound healing (in vivo)	Zhu et al. (2020)
	ALG-MA	Cryogel (injectable)	Chemoimmunotherapy (in vivo)	Bauleth-Ramos et al. (2019)
	Physical crosslink (CaSO4)	Hydrogel	Drug delivery	Kennedy et al. (2016)
	Physical crosslink (CaSO4)	Hydrogel	Drug delivery	Kearney et al. (2015)
	Physical crosslink (CaCl2)	Hydrogel	Drug delivery for wound healing	Kadri et al. (2021)
FU	N.A.	Hydrogel	Wound healing (in vivo)	Shanmugapriya et al. (2020)
	FU and CS ECH crosslinking	Hydrogel (patch)	Oral inflammatory diseases	Zheng et al. (2021)
	FU functionalization on PVA	Hydrogel	Endothelialization (In vivo)	Yao et al. (2020)
	N.A.	Hydrogel	Cartilage TE	Carvalho et al. (2022)
	ECH crosslinking	Hydrogel	Scaffolds for TERM	Hao et al. (2021)
LAM	LAM-MA	Hydrogel (injectable)	Biomedical platform	Custódio et al. (2016)
	LAM-MA	Hydrogel	Regeneration devices, disease models, and cell delivery systems (in vivo)	Zargarzadeh et al. (2022)
	LAM-ALD	Hydrogel	Biomedical applications	Lavrador et al. (2020)
	LAM-Boronic acid	Hydrogel	TE	Amaral et al. (2020)
	LAM-Boronic acid	Hydrogel	Biomedical platforms	Amaral et al. (2021)
k-CG	Poly(acrylic acid)-graft-κ-CG	Hydrogel	Drug delivery	Bardajee et al. (2020)
	N.A.	Hydrogel (injectable)	Biomedical platform	Li et al. (2018)
	N.A.	Hydrogel	Scaffolds for TERM (In vivo)	Qi et al. (2020)
	N.A.	Hydrogel (injectable)	Mandibular distraction (In vivo)	Wang et al. (2010)
	Physical crosslink (KCl)	Hydrogel beads	Biomedical applications	Azizi et al. (2017)
Agar	Free-radical crosslinking of agar and κ-CG using tri (ethylene glycol) divinyl ether (TEGDE)	Hydrogel	Wound dressing	Polat et al. (2020)
AGR	N.A.	Ionogel ([HEA][HCOO] or its mix with [C4mim][Cl])	Soft matter electronic devices and biomedical applications	Trivedi et al. (2012)
	N.A.	Ionogel ([C4mim][Cl)	Wound dressing, electronic devices or drug delivery	Trivedi et al. (2013)
	N.A.	Hydrogel	Cartilage tissue regeneration	Ahearne and Kelly (2013)
	N.A.	Hydrogel	Biomedical applications	Moghassemi et al. (2017)
	N.A.	Hydrogel	Drug delivery	Marras-Marquez et al. (2015)
	N.A.	Hydrogel	Protein delivery for axonal growth	Mehrotra et al. (2010)
	N.A.	Hydrogel	Controlled drug release	Lynam et al. (2014)

**FIGURE 5:**
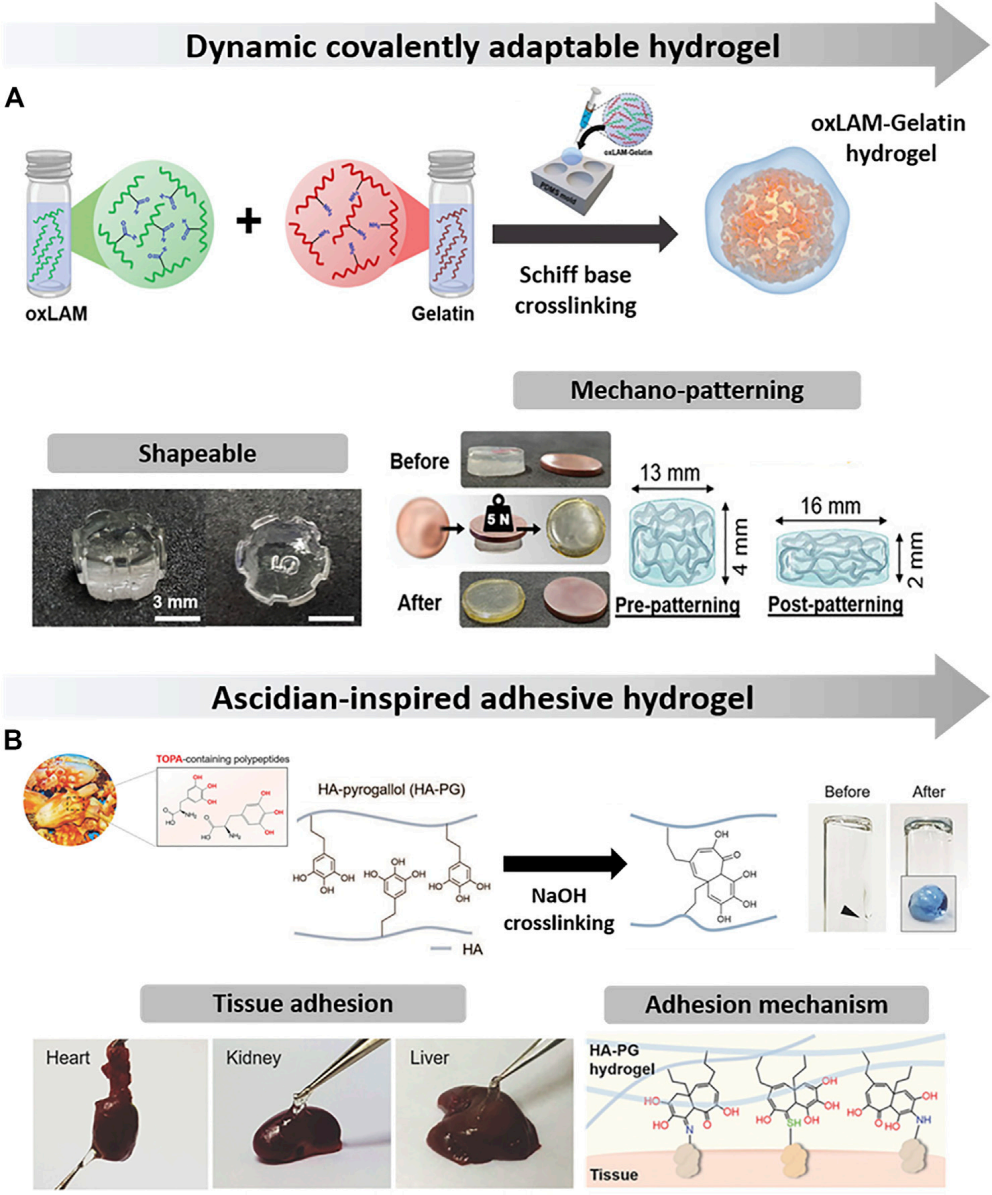
**(A)** Dynamic covalently adaptable hydrogels imprinted with an anisotropic nanotopographical configuration where cells have aligned along the intercalating nanoridge topography. Adapted from (Lavrador et al., 2020) with permission from John Wiley & Sons, Copyright 2020 WILEY-VCH Verlag GmbH & Co. KGaA, Weinheim; **(B)** Ascidian-inspired gallol-modified hyaluronic acid hydrogel adhered to various organs through covalent binding of gallol moieties with nucleophilic groups present at tissues interface. Adapted from (Cho et al., 2018) with permission from John Wiley & Sons, Copyright 2017 WILEY-VCH Verlag GmbH & Co. KGaA, Weinheim.

Although providing an adequate microenvironments to guide cells, conventional polysaccharide hydrogel-networks have been widely applied as passive and static 3D supports for embedded cells, thus lacking the innate ECM viscoelasticity and its highly dynamic behavior that enables matrix remodeling (Neves et al., 2020). Aiming at recapitulating such features, innovative chemistry toolboxes have been actively investigated toward the design of ECM biomimetic networks (Kirschning et al., 2018; Jiang et al., 2019). Among available chemical strategies, incorporating dynamic bonds into marine polysaccharide hydrogels has been emerged as one of the most attractive approach to recapitulate specific ECM functionalities. In this regard, viscoelastic and adaptable hydrogels comprising laminarin chemically modified with pendant aldehyde moieties and gelatin were engineered *via* Schiff base crosslinking chemistry (Figure 5A) (Lavrador et al., 2020). Adjusting aldehyde-to-amine ratios afforded a fine-tuned control over crosslinking kinetics, viscoelastic properties, and degradability profile. Furthermore, complex topographies were able to be imprinted on post-crosslinked hydrogel through mechanical imprinting process due to the covalently adaptable hydrogel networks that underwent spatially rearrangement during compressive loading.

The incorporation of dynamic bonds into hydrogels also leverages on-demand hydrogel functionalities as self-healing and/or shape-memory properties with peculiar interest on injectable tissue engineering strategies (Mehrali et al., 2017). Inspired on the mussel byssus adhesiveness, a robust marine polysaccharide-based hydrogel was fabricated by combining a chemically crosslinked chitosan-based network with dynamic non-covalent bonds through metal coordination of iron (III) cations with pendant catechol moieties present into the CHT backbone (Azevedo et al., 2017). Such physical crosslinking has enabled efficient energy dissipation as sacrificial bonds upon compressive deformation and, ultimately, excellent toughness, fast self-recovery capability, good fatigue resistance, shear-thinning and self-healing properties. Besides coordination bonds capability, catechol and other phenolic groups also endow polysaccharide-based hydrogels with outstanding self-adhesive properties on wet conditions, thus making them suitable as a surgical sealant or glue for sutureless wound closure (Kim et al., 2020; Li et al., 2020; Zhou et al., 2020). The mechanism behind tissue adhesion mainly relies on the strong binding affinity of oxidized catechol moieties towards different nucleophilic groups (e.g., amine, thiol and imidazole) of peptides/proteins found at tissues interface (Kim et al., 2020). So, catechol- and gallol-grafted hyaluronic acid hydrogels have showcased strong adhesion onto the surface of various organs (Shin et al., 2015; Cho et al., 2018), including liver, heart and kidney (Figure 5B), remaining tightly bound for up to 2 weeks. Considering the strong tissue adhesiveness of these mussel-inspired hydrogels, they could be also applied as delivery therapeutic cells without injection or invasive procedures (Cho et al., 2018; Koivusalo et al., 2019). For instance, human adipose-derived stem cells (hASCs) encapsulated in catechol-grafted hyaluronic hydrogel were directly painted onto the infarcted area of rat hearts, providing an effective injection-free cell transplantation into the ischemic myocardium (Shin et al., 2015). Owing to their high affinity to nucleophiles, mussel-inspired polysaccharide-based hydrogels can also immobilize various growth factors and bioactive molecules that would impact cellular behavior and functions. It was found that incorporating basic fibroblast growth factor (bFGF) into catechol-grafted hyaluronic acid hydrogels increased the viability and hepatic function of human hepatocytes without a loss of loaded biomolecules over 2 weeks (Shin et al., 2015), thus indicating that the embedded growth factors could be chemically conjugated to hydrogel catechol moieties rather than being physically entrapped within it (Lee et al., 2013).

Recently, self-healable hydrogels have also been employed as supporting baths for embedding shear-thinning hydrogel inks (Highley et al., 2015; Hoon Song et al., 2018). Both hydrogels were assembled through guest–host interactions by functionalizing HA with guest–host pairs of adamantane (Ad) (Ad-HA) and β-cyclodextrin (β-CD) (CDHA). Additionally, in one of those studies (Hoon Song et al., 2018) the support hydrogel was further reinforced with norbornene (Nor) (AdNor-HA) to be covalently crosslinked through a thiol-ene reaction. Such chemistry facilitated the stabilization of the support hydrogel, while removing the self-healable hydrogel ink by disrupting the guest–host bonds with a solution containing excess β-CD. Such approach has been particularly attractive for printing complex and freestanding 3D constructs with open channels. Aside from providing the stabilization of supporting bath, norbornene pendant moieties have also allowed the further anchorage of arginine–glycine–aspartic acid (RGD) peptides, enabling the endothelial cells’ seeding and their sprouting into the supporting hydrogel, which underwent on-demand protease-sensitive degradation when cells were exposed to angiogenic factors. The formation of confluent cell monolayers within complex and different blood vessel-shaped microchannels hold great potential for engineering vascular grafts.

One should be mentioned that the use of cyclodextrin and adamantane guest–host interactions has also been advantageous to self-assembly hyaluronic-based microgels, affording the formation of granular hydrogel systems (Mealy et al., 2018). In particular, such guest-host interparticle crosslinking have imparted shear-thinning and self-healing properties to the granular hydrogel, rendering it amenable to injection into dynamic environments. The possibility of combining microgels with varied compositions into a single granular hydrogel hold great potential for the therapeutic design of hydrogels with multiplex functionalities.

Other recent strategy to design functional and perfusable vascular networks for embedding into large-scale hydrogel constructs, wherein nutrient/oxygen diffusion limitations compromised cellular viability into the hydrogel core region, involves the synergistic combination of the 3D printing and the layer-by-layer (LbL) assembly technologies (Sousa et al., 2021). In such approach, alginate was employed as ink to produce customizable 3D sacrificial microstructures that were further coated with bioinstructive CHT and RGD-coupled ALG multilayers *via* LbL assembly. After embedding the LbL-coated ALG structures into a shear-thinning photo-crosslinkable supporting hydrogel, the alginate core template was liquefied giving rise to perfusable microchannels wherein the multilayered membrane shell acts as physical selective barrier, thus mimicking the native basement membranes that surrounded most mammalian tissues. This work was an interesting approach to recapitulate the native 3D microenvironment where cells reside *in vivo*.

With the significant advances on the hydrogels’ design, new classes of hydrogel-based biomaterials have been emerged such as cryogels (Eggermont et al., 2020) and ionogels (Luo et al., 2022). Cryogels are injectable preformed scaffolds with shape–memory properties, typically formed in water solvent with controlled polymerization subzero temperatures (Jones et al., 2009). Such conditions affords the formation of sponge-like structures with highly interconnected macroporous network that facilitates cellular infiltration, cell organization and proliferation, thus being suitable for TERM applications, drug delivery and immunotherapy (Memic et al., 2019). Recently, covalently crosslinked cryogels made of methacrylated-hyaluronic acid functionalized with RGD domains were engineered to withstand autoclave sterilization, which has substantially improved the cryogel biocompatibility after subcutaneous injection, with minimal induced immune reaction at the implantation site (Villard et al., 2019).

Concerning ionogels, they are polymeric networks that are swollen with ionic liquids (ILs). In particular, the use of ILs with polysaccharides are attractive to endow them with electrical properties towards the development of electroactive tissues (e.g., cardiac, skeletal, muscle, brain, and nerve). For instance, VPImBF4 ionic liquid was grafted onto methacrylated-chitosan upon UV-light exposure, enabling the formation of an electroactive hydrogel, which conductivity matched the one reported for spinal cord tissue (Wang et al., 2022). Therefore, such ECM-like electroactive hydrogel has enhanced the neuronal differentiation response, also triggering the neovascularization. Such ionogel offers a new approach to engineer electroactive implants for spinal cord injury repair.

## Sustainability issues on the sourcing of raw materials

The bioeconomy paradigm aims to foster a sustainable and circular economy supported by renewable biological resources. However, the growing interest on the use of marine organisms for a multitude of high-end uses may impact involuntarily their natural populations and negatively affect the marine ecosystems. Even marine taxa which have been explored for several decades are now being more closely monitored and their collection from the wild more strictly regulated. This is, for example, the case of Gelidium, the highly prized red seaweed used to produce Agar since the end of the 1800s. When mixed with water, this gelatinous substance forms hard gels and is used around the world by biologists to culture microorganisms. The growing concern with the conservation status of natural populations has led authorities from the main supplying countries (e.g., Morocco and Spain) to restrict the harvest of Gelidium. With no signs for the global demand for Agar to slow down in upcoming years, with no unexplored wild populations of Gelidium available to supply algal biomass and with its production through aquaculture not being technically nor economically feasible, prices for this polysaccharide have skyrocketed.

**FIGURE 6:**
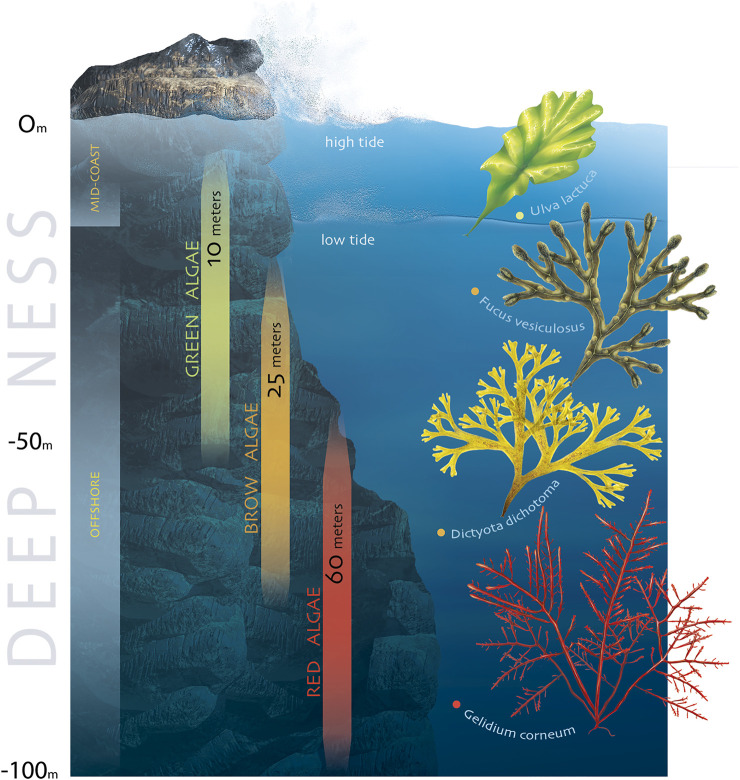
Distribution of main seaweed species in coastal waters.

The scenario described above for Gelidium may eventually also occur for other seaweeds currently harvested from the wild and whose highly prized polysaccharides may also experience an increase in price. From brown seaweeds yielding FU (e.g., Fucus), ALGs and LAM (e.g., Laminaria and Saccharina), to red seaweeds sourced for k-CG (e.g., Chondrus and Eucheuma) and green seaweeds to produce UL (e.g., Ulva), their natural populations may soon be unable to cope with the high demand if aquaculture is unable to boost supply. Seaweeds biodiversity is mirrored by their chemodiversity (Leal et al., 2013), as their life history allowed them to colonise different habitats along the depth gradient. While green seaweeds commonly occur in shallower waters, as their photosynthetic pigments are less efficient harvesting light wave lengths that can penetrate deeper in the water column, brown seaweeds have evolved to colonise deeper waters. Non-etheless, red seaweeds were the ones that have evolved to better harness the light wave lengths that can still penetrate to the lower limit of the photic zone (Figure 6). If one focus in Europe, it is possible to see that over the last two decades the harvesting of seaweed populations from the wild continued to be the primary production source for macroalgal biomass. It is therefore urgent to boost aquaculture production to keep-up with demand (Araújo et al., 2021). While offshore aquaculture is the one that presents a higher potential for scaling up production, land-based aquaculture allows to have a much higher control over the quality of algal biomass being produced, as well as their potential contamination by a multitude of pollutants. While certainly more costly than offshore aquaculture, land-based production when performed under bio-secure protocols can deliver algal biomass that fetches significantly higher values in premium cosmeceutical, nutraceutical, and biomedical markets. Indeed, the higher the position in the value chain of the biomolecules being sourced, the more likely it is that aquaculture of the species yielding them is commercially feasible (Araújo et al., 2021).

Several polysaccharides of commercial relevance are also sourced from marine animals. For instance, CHT is mostly derived from the exoskeletons of decapod crustaceans, namely shrimps. Non-etheless, most shrimp exoskeletons employed for this purpose result from the peeling processing of specimens sourced from fisheries for human consumption (e.g., the northern prawn Pandalus borealis) or farmed in Asian countries (e.g., whiteleg shrimp *Penaeus* vannamei). This is an excellent example of a circular bio-based approach that aims to add value to a side-stream (the exoskeletons) of a perfectly well-established value chain associated with the global processing of wild and farmed shrimp (De Aguiar et al., 2021). For example, PRIMEX is a recognized Icelandic marine biotechnology company, which is leader in processing shrimp shells raw-materials of Pandalus borealis into high quality CHT powder and based products for use in medical devices and personal care. Currently, PRIMEX product portfolio comprises CHT capsules for effective weight management (LIPOSAN), CHT medical spray and gel for natural healing (ChitoCare), CHT pool clarifier (SeaKlear) and animal CHT gel and spray for damaged skin as well as chronic wounds and ulcers (ChitoClear).

Also, the use of side streams from the processing of farmed fish, such as Atlantic salmon (*Salmo salar*), is certainly a sustainable way to obtain some polysaccharides of commercial relevance. For instance, while producing hyaluronan from the bacteria *Streptococcus* zooepidemicus remains as the most economically appealing approach (Chen et al., 2019), marine derived hyaluronan can be sourced from farmed Atlantic salmon (Kakizaki et al., 2017). This polysaccharide can also be yielded from the eye bulbs of several fish species targeted by commercial fisheries (e.g., swordfish, Xiphias gladius). This type of biorefinery approach is paramount to foster circularity and safeguard that non-edible parts (eye bubs, bones, skin, viscera … ) of marine living resources sourced from fisheries are used to its full potential. HA can also be sourced from other marine organisms, but the sustainability of such approach is certainly more questionable, as cnidarians and echinoderms are less likely to endure high fishing pressure. This premise is particularly relevant for sharks and rays, which can also yield marine HA, as well as CS. This last polysaccharide has been signalled from several species whose conservation status are of significant concern. This is the case of the shortfin mako shark (*Isurus oxyrinchus*) or the blue shark (*Prionace glauca*), whose populations worldwide are facing an unprecedented level of fishing pressure that is rapidly pushing them towards extinction (Dulvy et al., 2008). While other sharks and rays body parts can also yield CS, this polysaccharide has been often reported from the cartilages of shark fins. Shark fins are often obtained through a dreaded and illegal practice called finning–the dorsal, pectoral, and caudal fins of sharks are cut onboard, and specimens are thrown back to the sea still alive, where they suffer a slow and painful end, either by drowning or bleeding to death. The use of side streams from the processing of sharks sourced from commercial fisheries may be a solution, but its sustainability may only be valid if the fishery itself is sustainable. It is also worth referring that, non-governmental organizations and social media, are particularly active in exposing the cruelty of shark finning and banning the capture of several emblematic shark species already signalled in the Red List of Threatened Species by the International Union for Conservation of Nature (IUCN). Consumers of beauty and health-related products that incorporate marine origin polysaccharides are increasingly aware of sustainability issues, particularly those in the western markets of the northern hemisphere. Consequently, such products incorporating marine polysaccharides derived from sharks, rays and other marine species known to be endangered may rapidly become unpopular and be less appealing for high-end commercial uses.

Overall, while algae-derived marine polysaccharides will likely experience a growing acceptance by consumers that commonly perceive them as sustainable, this may not exactly be the case for those derived from marine animals, namely those originating from flagship species for marine conservation, such as sharks. With the forecasted growth of marine seaweeds and finfish aquaculture, along with increasing concerns to properly manage world fisheries, more sustainably sourced biomass will be available to yield marine polysaccharides of commercial relevance. The sustainable use of marine polysaccharides can contribute to the 2030 United Nations Agenda for Sustainable Development, namely to the Sustainable Development Goal (SDG) 3 (Good Health and Wellbeing), by yielding health promoting biomolecules, as well as to SDG 12 (Responsible Consumption and Production), by fostering biorefinery approaches aligned with a circular bioeconomy, that allow to fully use marine resources from fisheries and aquaculture sourced for human consumption. Ultimately, by adding value to marine chemodiversity, the use of marine polysaccharides can raise awareness towards the need to preserve marine biodiversity and contribute to achieve SDG 14 (Life Bellow Water). Marine biomass produced under high-biosecurity standards, as well as that sourced from side-streams from the transformation industry of seaweeds and marine seafood will likely become the cornerstone of marine polysaccharides value-chains, in line with the Blue Bioeconomy framework currently advocated.

## Overview and future perspectives

The marine environment has proven to be an extraordinary source of bioactive molecules for a multitude of biomedical and biotechnological applications owing to their renewable character and very appealing physicochemical and biological properties. As such, marine-origin polysaccharides extracted from marine biomass are a highly valuable alternative to polymers obtained from petroleum sources, having a huge impact on the preservation of the environment, wellbeing of society, and the sustainability of our Planet. In fact, to date, marine biopolymers have already ignited a great interest in several commercial applications in different fields such as food, cosmetics, packaging, or biomedicine. However, when massively used, a major burden is imposed in their natural populations and marine ecosystems. Also, the chemical strategies most commonly employed to enable the extraction of marine polysaccharides encompass the use of hazardous chemicals and the generation of hazardous by-products, often being disposed, together with the marine biomass waste, into and contaminating the ocean, which imposes a major burden to the natural ecosystem and human health.

In this regard, the continuous exploitation of efficient, cost-effective, environmentally-friendly and sustainable methodologies for enabling and valorising the efficient extraction of polysaccharide from marine biomass to produce added-value biomaterials for biomedicine is of upmost importance to address the United Nations Sustainable Development Goals (UN SDGs) and enabling a sustainable future globally.

In this context, we would suggest some guidelines for the valorisation of marine biomass as high-added value products for healthcare applications: i) establish efficient, simple and eco-friendly extraction processes that could be applied in pilot plants; ii) unlock multiple functionalities for marine polysaccharides by combining computational and synthetic green chemistry towards the man-designed advanced biomaterials; iii) pursuit advanced research to exploit their full market potential encompassing scientists’ collaboration from interdisciplinary fields; and iv) promote synergistic interactions between academia and industries to foster marine-derived biomaterials into ready-to-use market products.

## Glossary

AAC: Azyde-alkyne cycloaddition
AGR: Agarose
ALG: Alginate
CC: Click-chemistry
CHT: chitosan
CS: chondroitin sulfate
DES: Deep eutectic solvent
DIC: N,N′-diisopropylcarbodiimide
ECM: Extracellular matrix
EE: Enzymatic extraction
EDC: 1-ethyl-3-(3-dimethyl-aminopropyl-1-carbodiimide)
FU: Fucoidan
GAG: Glycosaminoglycan
HA: Hyaluronic acid
ILs: Ionic liquids
k-CG: Carrageenan
LAM: laminarin
LbL: Layer-by-layer
MAE: Microwave-assisted extraction
NHS: N-hydroxysuccinmide
PLE: Pressurized liquid extraction
RGD: Arginine-glycine-aspartic acid
RM: Regenerative medicine
SDG: Sustainable Development Goal
SPAAC: Strain-promoted azyde-alkyne cycloaddition
SWE: Subcritical water extraction;
TE: Tissue engineering
TERM: tissue engineering and regenerative medicine
UAE: Ultrasound-assisted extraction
UL: Ulvan
UN SDGs: United Nations Sustainable Development Goals.
